# Supplementary figures and images for: MAPK pathway mutations in head and neck cancer affect immune microenvironments and ErbB3 signaling
Source: Life Sci Alliance. 2020 May 7;3(6):e201900545. doi: 10.26508/lsa.201900545 (PMC7219112; doi:10.26508/lsa.201900545)

Source Data – Figure 2b

Upper panel

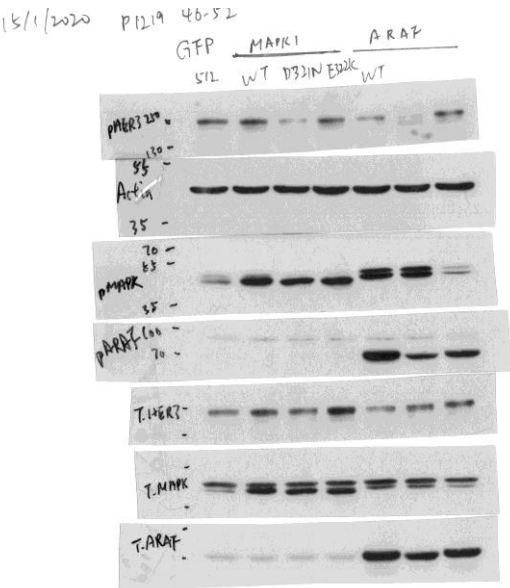

Lower panel

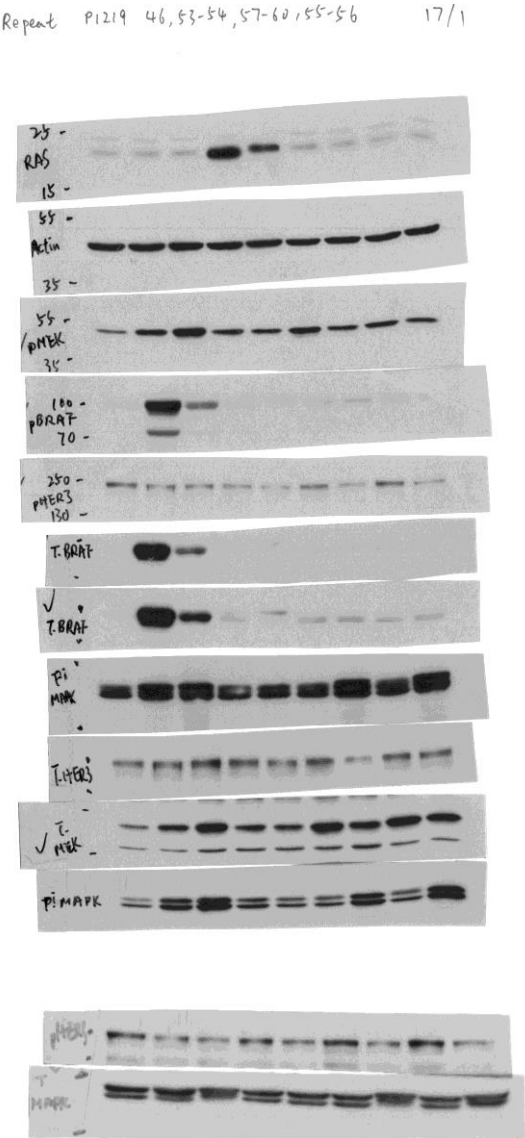

Supplement: Supplementary file 5 [file LSA-2019-00545_SdataF2B.pdf]
